# Assessment of patient-derived tumour xenografts (PDXs) as a discovery tool for cancer epigenomics

**DOI:** 10.1186/s13073-014-0116-0

**Published:** 2014-12-12

**Authors:** Paul Guilhamon, Lee M Butcher, Nadege Presneau, Gareth A Wilson, Andrew Feber, Dirk S Paul, Moritz Schütte, Johannes Haybaeck, Ulrich Keilholz, Jens Hoffman, Mark T Ross, Adrienne M Flanagan, Stephan Beck

**Affiliations:** Medical Genomics, UCL Cancer Institute, University College London, London, WC1E 6DD UK; Genetics and Cell Biology of Sarcoma, UCL Cancer Institute, University College London, London, WC1E 6DD UK; Department of Biomedical Sciences, University of Westminster, London, W1W 6UW UK; Translational Cancer Therapeutics Laboratory, CR-UK London Research Institute, London, WC2A 3LY UK; Alacris Theranostics GmbH, 14195 Berlin, Germany; Institute of Pathology, Medical University of Graz, 8036 Graz, Austria; Department of Hematology and Medical Oncology, Charité Comprehensive Cancer Center, D-10117 Berlin, Germany; EPO-Berlin-Buch GmbH, 13125 Berlin, Germany; Illumina Cambridge Ltd, Chesterford Research Park, Little Chesterford, CB10 1XL UK; Department of Histopathology, Royal National Orthopaedic Hospital NHS Trust, Stanmore, Middlesex, London, HA7 4LP UK

## Abstract

**Background:**

The use of tumour xenografts is a well-established research tool in cancer genomics but has not yet been comprehensively evaluated for cancer epigenomics.

**Methods:**

In this study, we assessed the suitability of patient-derived tumour xenografts (PDXs) for methylome analysis using Infinium 450 K Beadchips and MeDIP-seq.

**Results:**

Controlled for confounding host (mouse) sequences, comparison of primary PDXs and matching patient tumours in a rare (osteosarcoma) and common (colon) cancer revealed that an average 2.7% of the assayed CpG sites undergo major (Δβ ≥ 0.51) methylation changes in a cancer-specific manner as a result of the xenografting procedure. No significant subsequent methylation changes were observed after a second round of xenografting between primary and secondary PDXs. Based on computational simulation using publically available methylation data, we additionally show that future studies comparing two groups of PDXs should use 15 or more samples in each group to minimise the impact of xenografting-associated changes in methylation on comparison results.

**Conclusions:**

Our results from rare and common cancers indicate that PDXs are a suitable discovery tool for cancer epigenomics and we provide guidance on how to overcome the observed limitations.

**Electronic supplementary material:**

The online version of this article (doi:10.1186/s13073-014-0116-0) contains supplementary material, which is available to authorized users.

## Background

Xenografting of human tumours into mice or rats has been performed since the late 1960s [1], but it was not until the advent of immunodeficient mouse strains (for example, severe combined immunodeficiency (SCID) mice) in the mid-1980s that the practice became widespread in basic research and preclinical studies [2]. These new models of disease brought with them new hopes of therapeutic advances but have also displayed a number of noteworthy limitations [2]. Firstly, both the surrounding stroma and the blood vessels recruited to the growing tumour during angiogenesis effectively incorporate murine cells into the transplanted tumour. Secondly, placing the xenograft orthotopically is technically challenging, so most are grown subcutaneously, effectively eliminating the possibility of replicating metastatic disease. Despite these limitations, patient-derived tumour xenografts (PDXs) have proven extremely accurate at predicting drug response in various cancer types [3], and have been used in numerous preclinical studies [4].

Osteosarcoma (OS) is the most common form of primary bone cancer, yet remains incredibly rare with an age-standardised incidence in the UK of 8 and 6 per million in males and females, respectively [5]. Thus, one of the major issues with the study of rare cancers such as OS is the scarcity of primary samples to analyse. This highlights the need for an accurate model of the disease and PDXs have been shown in multiple cancer types to better represent the genetic and gene-expression characteristics of tumours than *in vitro* cell lines [6]. Moreover, because OS presents most often in adolescents and young adults, who are less likely to enrol into clinical trials [7], patient recruitment can often take several years, thus enhancing the inherent jeopardy in drug selection for these trials. With this in mind, *in vivo* tumour models that most accurately replicate the patient’s condition are a crucial factor in experimental pharmacology.

PDXs constitute one such model that is widely used in preclinical research [8], and OncoTrack, the largest European public-private biomarker consortium which aims to develop novel biomarkers for targeted therapy [9], generated PDXs that were included here as an additional tumour type and an example of a common cancer (colon cancer (CC)). Despite the popularity of PDXs, only a few systematic studies have compared their fidelity to the original tumours from which they were derived. Nonetheless, the findings have been encouraging: in pancreatic cancer, for instance, gene expression patterns were faithfully retained in PDXs and the majority of the observed changes were associated with pathways reflecting the microenvironment [10], and in breast cancer less than 5% of genes showed variation in expression between PDXs and the corresponding primary tumour [11]. To our knowledge, however, only one systematic genomic profiling of patient tumours and PDXs is available in the literature: it shows that all copy number variants are maintained in PDXs, and that while xenografts do initially present a small number of single nucleotide variants (approximately 4,300), the vast majority of changes that accumulate over time occur in non-coding parts of the genome [12]. Similarly, only one study has assessed genome-wide DNA methylation changes in head and neck squamous cell carcinomas using the earlier Infinium 27 K BeadChip, and found no statistically significant changes [13].

To address this gap in our current knowledge, we have carried out a comprehensive assessment of the suitability of PDXs for cancer epigenomics. The assessment included methylome analysis using array- and sequencing-based technologies of primary and secondary PDXs derived from rare (OS) and common (CC) cancers as well as computational simulations.

## Methods

### Tumour samples and xenografting

The research described below conformed to the Helsinki Declaration.

For OS, PDXs were generated from tumour samples obtained from the Stanmore Musculoskeletal Biobank, satellite to the UCL Biobank for Health and Disease. Ethical approval for the OS samples was obtained from the Cambridgeshire 2 Research Ethics Service, UK (reference 09/H0308/165), and the UCL Biobank for Health and Disease, which is held under the Human Tissue Authority licence 12055: project EC17.1. Samples were washed in phosphate-buffered saline and cut to the appropriate size (approximately 2 to 3 mm^3^). Under isoflurane anaesthesia delivered via a nasal attachment tube, tumour fragments were inserted subcutaneously in one or both flanks of the mice. In total, 14 female SCID mice (3 to 6 weeks old) were kept at the UCL Animal Housing facility in individually ventilated cages, and monitored at least twice a week for the duration of the experiment. Procedures were followed as described in the project license (delivered by the UK Home Office PPL 70/6666) and, when necessary, animals were sacrificed according to an approved schedule 1 protocol. Tumour growth was measured using digital measuring callipers. Tumours were snap-frozen in liquid nitrogen after excision.

For CC, PDXs were generated from tumour tissue derived from surgical specimens of patients with colorectal cancer. The tissue samples and respective data from the Medical University of Graz were provided by the Biobank Graz with Ethics approval of the project under the ethical commission number 23-015 ex 10/11. The tissue samples and data from the Charite Medical University in Berlin were provided with Ethics approval EA1/069/11. The tumour samples were received directly from the hospitals in Berlin (Charité) and Graz (Medizinisches Universitätsklinikum) under sterile conditions. The tumours were cut into 2 × 2 mm fragments and placed in a sterile Petri dish covered with HANKs balanced salt solution. Mice were anaesthetised by a single intravenous injection (0.15 ml/mouse) with Etomidate-®Lipuro (0.3 mg/mouse) and each fragment was inserted subcutaneously into the left flank of the recipient mouse. We used immune deficient female NMRI nu/nu mice, supplied from Taconic (Lille Skensved, Denmark) or Charles River (Sulzfeld, Germany). The mice were kept at EPO under sterile conditions in strictly controlled and standardised barrier conditions, IVC System Tecniplast DCC (Tecniplast Deutschland GmbH, Hohenpeißenberg, Germany). The body weight and health of the mice were controlled throughout the experiment. After the xenotransplantation, tumour growth was monitored at least twice a week using callipers. Mice were sacrificed when the tumours reached a volume of ≥1,000 mm^3^ or when the animals lost ≥20% body weight.

### DNA methylation analysis

Genomic DNA was extracted from PDX samples using the QIAamp DNA Mini Kit (Qiagen, Venlo, Limburg, Netherlands) according to the manufacturer’s instructions, and subjected to methylation analysis. The bisulphite conversion of the DNA was performed using the EZ DNA Methylation kit (Zymo Research, Irvine, California, USA) on 500 ng. Conversion efficiency was assessed by quantitative PCR. The Illumina Infinium HumanMethylation450 BeadChips were processed as per the manufacturer’s instructions. The MeDIP-seq libraries were prepared as previously described [14] and sequenced on a HiSeq 2000. The resulting 450 K and MeDIP-seq data were deposited into the Gene Expression Omnibus as a SuperSeries under accession number GSE59352.

### Statistical analysis

The raw data obtained from the 450 K arrays were processed from the IDAT files through to normalisation with BMIQ [15] using the ChAMP [16] pipeline, and all subsequent analysis was performed with the R statistical software v3.0.2 and custom scripts. Quality control of the array data included removal of probes for which any sample did not pass a 0.01 detection *P*-value threshold, bead cutoff of 0.05, and removal of probes on the sex chromosomes. Probes passing the detection *P*-value threshold of 0.01 in the mouse-only sample were also removed from downstream analysis in all xenografts to avoid confounding signal from any mouse DNA. The genomic and epigenomic features used are those annotated on the array and enrichments were calculated on the basis of 1,000 repetitions of a random selection of probes from the overall probe set used in the analysis.

The sequencing data were processed from fastq files using the MeDUSA [17] pipeline. The reads were aligned separately to both the hg19 and mm10 genomes, with all redundant and unpaired reads removed. After assessment of the levels of likely contamination from mouse DNA as well as based on recommendations from other studies [18], those reads aligning only to human or to both human and mouse were kept for downstream analysis.

## Results and discussion

### Comparison of osteosarcoma PDXs and patient tumours

**Figure 1:**
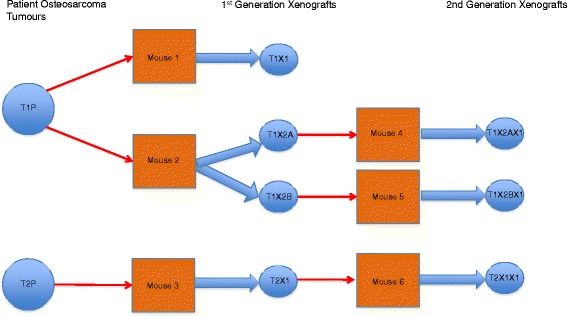
**Osteosarcoma PDX derivation scheme.** A single fragment from each patient tumour, approximately 1 mm in diameter, was inserted subcutaneously into each flank of a SCID mouse. Patient tumour 1 (T1P) gave rise to three first generation PDXs and two second generation PDXs, while patient tumour 2 (T2P) was used to produce one PDX at each generation.

To investigate the methylation changes linked to deriving xenografts from patient tumours, we subcutaneously inserted OS fragments from two patients in the flanks of SCID mice, and grew them over two generations according to the scheme described in Figure 1.

A final sample set consisting of two patient tumours (T1P and T2P), four first generation PDXs, and three second generation PDXs were available for methylation analysis on the Illumina Infinium 450 K Beadchips [19].

A major concern with analyses of human tumours grown in mice is the potential for signal contamination by host DNA from tumour vascularisation during its development or from the surrounding stroma when extracting the tumour. In order to eliminate these confounders in our methylation analysis, an additional mouse kidney sample was processed on the 450 K array and the 45,934 probes passing a detection *P*-value threshold of 0.01 were removed from downstream analysis. The use of the detection *P*-value ensured that probes were filtered out based solely on their ability to hybridise to the sample DNA as opposed to their methylation status. This makes the mouse kidney sample an appropriate tissue for filtering probes in the analysis of both types of cancers described in this study. The raw data for all samples were subsequently processed through the ChAMP analysis pipeline [16] (see Methods) to produce a final dataset of 9 samples and 463,558 probes.

**Figure 2:**
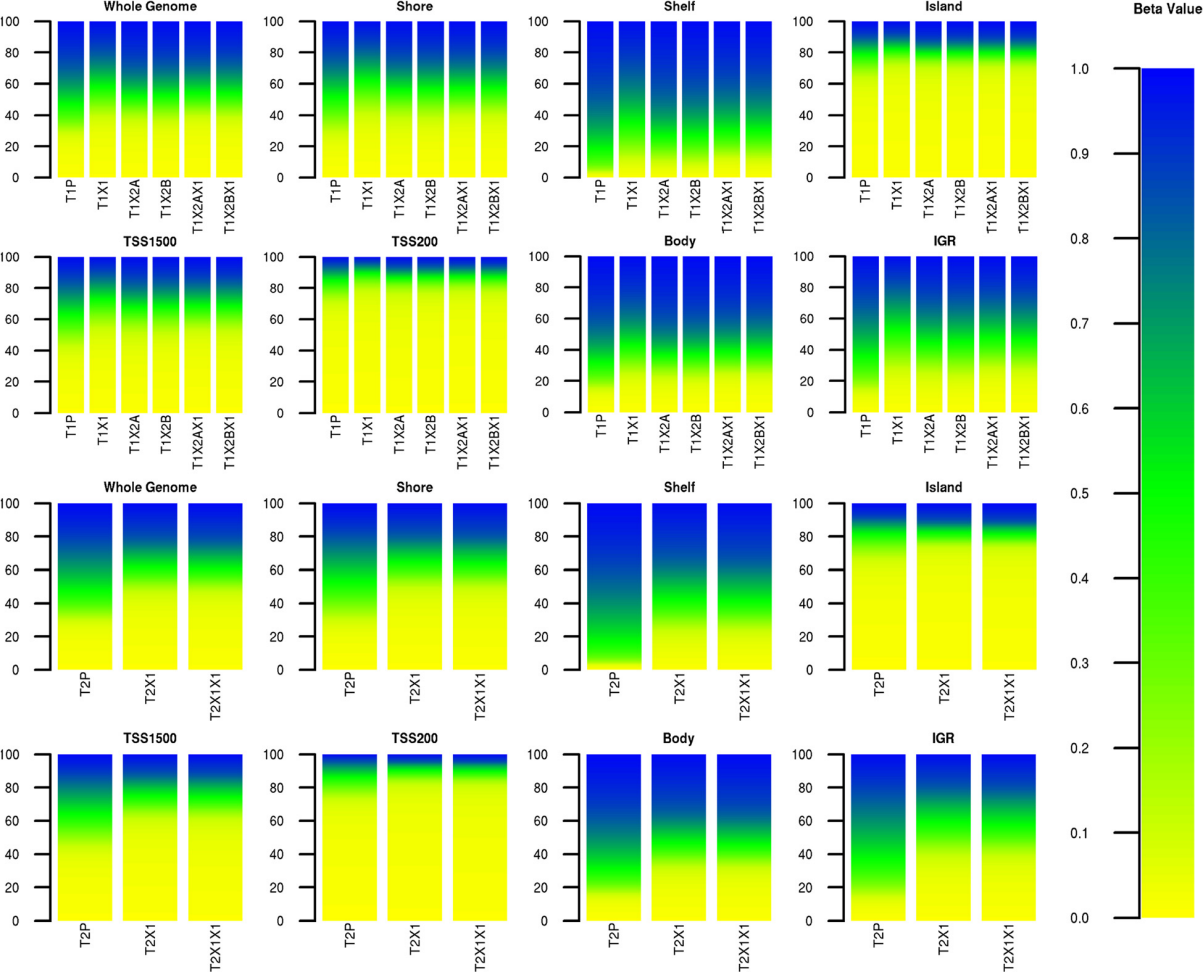
**DNA methylation distribution by feature.** For each feature, in each sample, the β-values are binned into 1% methylation increments (described by the colour scale), and the percentage of probes at each methylation level is shown in the individual plots. The top and bottom eight plots correspond to the T1 and T2 sets, respectively. Whole Genome = all probes. IGR, intergenic region; TSS, transcription start site.

The distributions of methylation at the genome-wide and feature-specific levels for each sample are shown in Figure 2. Although methylation levels appear remarkably consistent within each tumour set, and in line with expected feature-specific values (for example, low methylation at CpG islands), there is a slight increase in methylation levels across all features between the two patient tumours and their derivatives.

**Figure 3:**
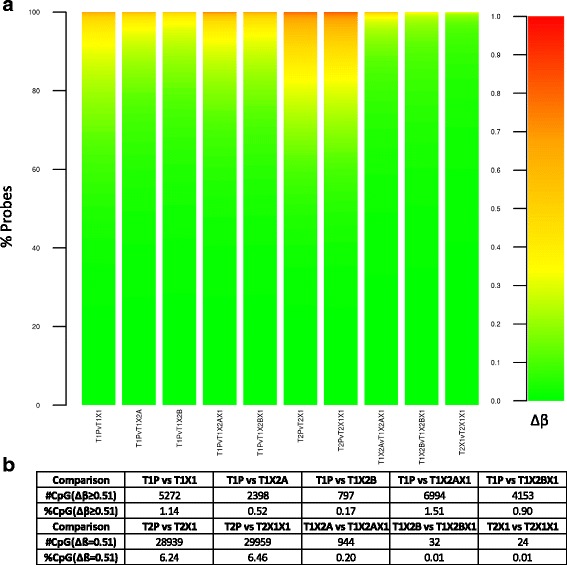
**Assessment of methylation changes in OS PDXs. (a)** For each PDX, the absolute difference (β_Patient_ - β_Xenograft_) is calculated at each probe and binned into 1% methylation difference increments (described by the colour scale); the percentage of probes showing each methylation difference level is shown in the individual plots. **(b)** Number and percentage of probes in each comparison changing by 0.51 or more, corresponding to all probes going from fully unmethylated to fully methylated and vice versa.

Specifically assessing methylation differences at each probe between a PDX and its original patient tumour further supports the maintenance of most of the methylome in tumour xenografts: Figure 3a shows that only a small fraction of the assessed CpG sites display large changes in methylation. We have previously shown [20] that 95% of fully unmethylated probes display β-values ≤0.31, while fully methylated probes have β-values ≥0.82; thus a Δβ threshold of 0.51 can be used as the minimum change expected for a CpG to be observed as going from fully unmethylated to methylated or vice versa ('reversed methylation'). Using this threshold in the comparisons of PDXs and patient tumours, as shown in Figure 3b, an average of only 0.85% of probes in the T1 set (n = 5) and 6.35% in the T2 set (n = 2) are measured as reversing their methylation status, leading to inaccurate results if using the PDX as a proxy for the patient tumour.

### Comparison of osteosarcoma PDXs across generations

Interestingly, although each set of PDXs displays this shift with xenografting, a constant profile is then maintained within a xenograft lineage: T1X2A, T1X2B and their second generation tumours all displayed consistent levels across features (Figure 2), as did T2X1 and T2X1X1, demonstrating that although the change in host is linked to a slight increase in methylation levels, subsequent xenografting is not accompanied by additional changes. This is confirmed by the vastly reduced number of reversed methylation events observed between first and second generation PDXs as opposed to those identified within the first generation; Figure 3 reveals that an average of only 0.07% (n = 3) of CpG sites see their methylation scores increase or decrease by over 0.51 after the first generation. This result suggests either an initial reaction to the new host that is then preserved in further generations as the mice used were isogenic, or a loss of tumour heterogeneity as only a fragment of the initial patient sample was used for xenografting, or a combination of these two factors. The fact that loss of heterogeneity would be expected to persist in further generations as only a fragment of the grown tumour is transplanted at each passage, and that signal from new host stromal cells and vascularisation affect gene expression in specific pathways (such as extracellular matrix formation) [10] suggests that the observed epigenetic change is due primarily to implantation of the tumour into a new host.

### Validation with MeDIP-seq

In addition to the methylation arrays, the OS PDXs and patient samples were analysed by methylated DNA immunoprecipitation followed by low-coverage next-generation sequencing (MeDIP-seq) [21]. Alignment, filtering of reads, and calling of differentially methylated regions (DMRs) were performed using the MeDUSA pipeline [17]. In order to minimise read contamination by mouse DNA, the fastq files were aligned separately to the human and mouse genomes and those reads aligning only to mouse were removed from downstream analysis. With our data this approach yielded a nearly identical final read set as using the Xenome [18] protocol, designed specifically for xenograft sequencing data, with over 98% overlap in each sample. Final read counts aligning to human, mouse or both are shown in Additional file 1.

The MeDIP-seq DMRs identified across all seven patient tumour/xenograft comparisons overlapped with 48 probes present on the 450 K array; importantly, the directionality of methylation change between patient tumour and xenograft was 100% concordant between the two methods, with the same 22 gains and 26 losses of methylation identified in the PDXs.

**Figure 4:**
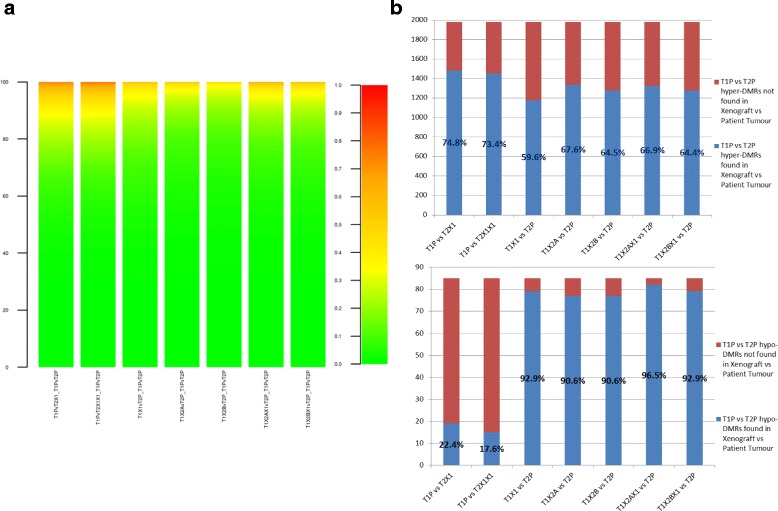
**PDXs as substitutes for patient tumours: 450 K versus MeDIP-seq. (a)** The absolute difference in β-value between the two OS patient tumours is calculated at each probe. The absolute difference between each PDX and the patient tumour from the other tumour set is then assessed, and a ΔΔβ for those two differences is calculated and plotted as in Figure 3. A result close to zero indicates concordance between the two measurements at a given CpG site. **(b)** Similarly to the process described above with the 450 K array, the number of DMRs between the two patient tumours that can be recapitulated between a PDX and the patient tumour are shown, for both hyper- and hypo-DMRs.

Similarly, in an inter-tumour comparison, when assessing the ability of a PDX to substitute for its matched patient tumour in an inter-tumour comparison (that is, T1P versus T2P), 450K and MeDIP-seq both identified similar trends (Figure 4): for each technology, the differences between the patient tumours T1P and T2P were assessed to act as a reference set; each PDX was then compared with the unmatched patient to see if the same differential methylation was captured. MeDIP-seq showed similar levels of concordance in the comparisons as the methylation array, with the exception of two of the hypomethylation sets (T1PvT2X1 and T1PvT2X1X1) that displayed lower levels of concordance (22.4% and 17.6%, respectively) in the MeDIP-seq data (Figure 4b). These, however, represent only small absolute differences in concordance (66 and 70 DMRs of the T1P versus T2P comparison were not identified in T1P versus T2X1 and T1P versus T2X1X1, respectively) due to the overall low number of hypomethylated DMRs detected between the two patient tumours (n = 85) compared with hypermethylated (n = 1,980).

### Methylome changes in colon cancer and osteosarcoma PDXs

**Figure 5:**
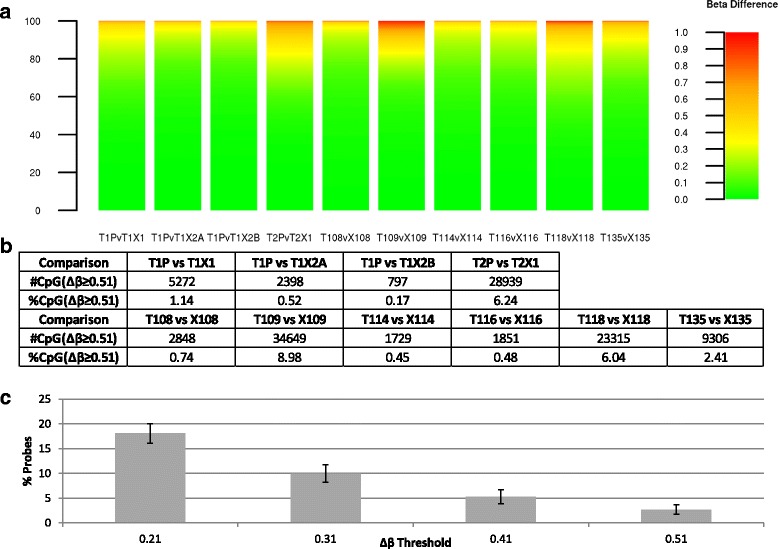
**Assessment of methylation changes in OS and CC PDXs. (a)** For each PDX, at each probe, the absolute difference (β_Patient_ - β_Xenograft_) is calculated and binned into 1% methylation difference increments (described by the colour scale); the percentage of probes showing each methylation difference level is shown in the individual plots. **(b)** Number and percentage of probes in each comparison changing by 0.51 or more, corresponding to all probes going from fully unmethylated to fully methylated and vice versa. **(c)** The mean percentage of changing probes across all samples at thresholds of 0.51, 0.41, 0.31, and 0.21. Error bars correspond to the standard error of the mean.

In order to further investigate those few CpG sites with changing methylation levels after xenografting, an additional set of six patient tumour/xenograft CC pairs from the OncoTrack consortium were assessed using Illumina 450K arrays and processed with the R package ChAMP. Grouping these with the first generation PDXs derived from OS tumours yields a final cohort of 10 sample pairs (Figure 5). Using the same Δβ threshold of 0.51 as for the OS samples, a similarly low number of probes were identified as changing with xenografting in the first generation, with an average of 3.18% (n = 6). Of note, when using lower Δβ thresholds, the average percentage of probes changing with xenografting increases to a maximum of 18% (Figure 5c).

To assess whether changes in methylation could be generalised to any tumour undergoing this procedure or whether they are tumour or tumour type-specific, the overlap in these changing probes within as well as between tumour types was evaluated. Statistically significant overlaps were found within each tumour type, with 236 probes changing in all first generation OS PDXs and five probes in CC PDXs (random resampling *P*-value <10^-4^); however, gene ontology tools (GREAT [22], Panther [23], DAVID [24]) did not reveal any particular functional links between these changing sites and no overlap was found between the two tumour types. This suggests that the changes in methylation observed with xenografting are unlikely to be due to a systematic reaction to the xenografting procedure but rather point to tumour specificity.

**Figure 6:**
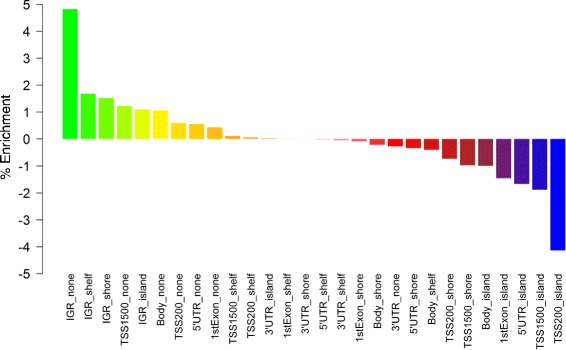
**Enrichment of (epi)genomic regions with changing methylation status after xenografting.** Each probe on the 450 K array is annotated to a genomic (TSS1500, Body, 3′ UTR…) and epigenomic (island, shore, shelf, none) region. These were combined for each probe to form a unique (epi)genomic annotation and enrichments were calculated using a random resampling strategy. IGR, intergenic region; TSS, transcription start site.

Finally, we assessed whether these methylation changes were more likely to occur in certain genomic and/or epigenomic features. As shown in Figure 6, these probes are depleted for promoter regions and CpG islands, but enriched for intergenic regions, particularly those with low CpG density (*P*-value <10^-4^).

**Figure 7:**
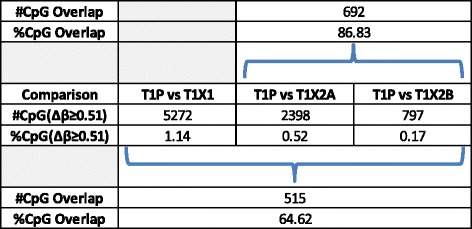
**Overlap of changing CpG sites between PDXs originating from the same patient tumour.** T1X2A and T1X2B were grown from T1P in two flanks of the same mouse. T1X1 was grown from T1P in a different animal. Overlap percentages were calculated based on the number of changing sites in T1X2B, the PDX with the fewest changes. Over 86% of probes changing in T1X2B also underwent major changes in T1X2A, and over 64% of changes were common between all three PDXs originating from T1P.

In the OS cohort, one of the patient tumours produced three first-generation PDXs, grown in two animals. Two of the PDXs (T1X2A and T1X2B) were harvested from the same mouse, one from each of the flanks. Despite the limited sample size, this set-up provides novel and important insights into the potential tumour specificity of the observed changes in methylation. The results displayed in Figure 7 reveal that over 86% of probes changing in T1X2B also underwent major changes in T1X2A, and over 64% of changes were common between all three PDXs originating from T1P. These overlaps, much higher than those observed within or across tumour types, further confirm tumour specificity of the observed methylation changes that accompany xenografting.

### Practical implications for the use of PDXs in epigenetic studies

**Figure 8:**
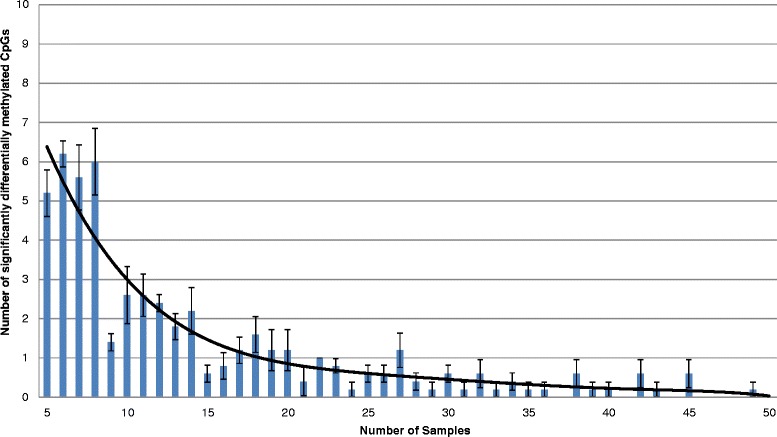
**Model of the effect of PDX-associated methylation changes.** For sample numbers *n* from 5 to 50, *n* random samples were randomly selected from 2,000 Marmal-aid data sets. Each sample was modified at 11,110 probes by β = 0.51 and a Wilcoxon rank-sum test run between the original n samples and the modified versions. The number of significantly differentially methylated probes (*P*-value ≤0.05) for each *n* is plotted against *n*. The model was run five times and the error bars represent the standard error of the mean at each *n*.

With a mean percentage of 2.7% (n = 11,110) of CpG sites undergoing major methylation shifts in first generation xenografts, PDXs appear to be more than adequate proxies for patient samples in methylation studies, as compared, for example, with the 0.27 to 0.72 correlation reported between whole blood and Epstein-Barr virus-transformed lymphocyte cell lines [25]. These are commonly used proxies in genetic studies, and have been previously used in associating methylation patterns with phenotypes [26]. However, the tumour-specific nature of these methylation changes implies that no accurate prediction as to which 2.7% of the measured methylation scores will be affected can reasonably be made beyond a general statement concerning enrichment in intergenic regions. In order to aid in the design of future studies, we devised a model to test how many 450K arrays should be run when comparing two groups of samples in order to minimise the effects of these tumour-specific xenografting-linked methylation changes. From a total of 2,000 data sets from Marmal-aid [27], a 450K data repository, we selected *n* (5 ≤ *n* ≤ 50) samples. These were taken at random from the tissue and disease types available to avoid any bias that might be introduced due to higher levels of similarity between the methylomes of samples from a particular tissue type compared with another. This ensures the resulting model can be used regardless of tissue origin. A total of 11,110 β-values in each sample were then increased or decreased by 0.51 (5,555 of each). We subsequently compared the original *n* samples from Marmal-aid with their modified counterparts and assessed the number of sites that appeared to be significantly differentially methylated between the groups (Figure 8), as determined by a Wilcoxon rank-sum test with a non-adjusted *P*-value threshold of 0.05. The non-adjustment for multiple testing allows flexibility in future study design, such as investigations using only a subset of the array.

This analysis revealed that the maximum number of probes significantly differentially methylated between the groups was eight, and if using 15 samples in each group, the xenografting-associated methylation changes will only significantly affect the differences between groups at two loci on average. This further demonstrates the suitability of tumour xenografts for methylome analysis. It is noteworthy that although using more than 15 samples in each cohort will continue to reduce the effect of xenografting-associated methylation changes on group methylation characteristics, the benefits in terms of affected probes will be substantially less than with the first 15 samples, as shown in Figure 8.

## Conclusions

This work advances our understanding of the epigenetic dynamics involved in PDX and provides guidance on the utility and interpretation of PDX-derived DNA methylation data. Our results from both rare (OS) and common (CC) cancer types show that less than 3% of the 450 K methylome undergoes major changes with xenografting. Moreover, these changes appear to be cancer-specific and little to no further methylation changes are observed in secondary xenografts. Finally, we propose a model to aid the design of future studies and minimise the impact of xenografting-associated confounding of DNA methylation in the interpretation of PDX-based studies.

## Additional file

Additional file 1: Table S1. Final MeDIP-seq read counts for osteosarcoma samples. The fastq files were aligned to both human (hg19) and mouse (mm10) genomes. Only reads that aligned to either human-only or both human and mouse were retained for downstream analysis.

## Abbreviations

CC: colon cancer
DMR: differentially methylated region
OS: osteosarcoma
PDX: patient-derived tumour xenograft
SCID: severe combined immunodeficiency
UTR: untranslated region
