# Supplementary material for: Assessment of patient-derived tumour xenografts (PDXs) as a discovery tool for cancer epigenomics
Source: Genome Med. 2014 Dec 12;6(12):116. doi: 10.1186/s13073-014-0116-0 (PMC4292812; doi:10.1186/s13073-014-0116-0)
Supplement: Additional file 1: Table S1. — Final MeDIP-seq read counts for osteosarcoma samples. The fastq files were aligned to both human (hg19) and mouse (mm10) genomes. Only reads that aligned to either human-only or both human and mouse were retained for downstream analysis. [file 13073_2014_116_MOESM1_ESM.doc]

**SUPPLEMENTARY DATA**

**Assessment of patient-derived tumour xenografts (PDXs) as a discovery tool for cancer epigenomics**

Paul Guilhamon^1^, Lee M. Butcher^1^, Nadege Presneau^2,3^, Gareth A. Wilson^1,4^, Andrew Feber^1^, Dirk S. Paul^1^, Moritz Schütte^5^, Johannes Haybaeck^6^, Ulrich Keilholz^7^, Jens Hoffman^8^, Mark T. Ross^9^, Adrienne M. Flanagan^2,10^, Stephan Beck^1*^

^1^Medical Genomics, UCL Cancer Institute, University College London, London, UK

^2^Genetics and Cell Biology of Sarcoma, UCL Cancer Institute, University College London, London, UK

^3^Department of Biomedical Sciences, University of Westminster, London, UK

^4^Translational Cancer Therapeutics Laboratory, CR-UK London Research Institute, London, UK

^5^Alacris Theranostics GmbH, Berlin, DE

^6^Institute of Pathology, Medical University of Graz, Graz, Austria

^7^Department of Hematology and Medical Oncology, Charité Comprehensive Cancer Center, Berlin, Germany

^8^EPO-Berlin-Buch GmbH, Berlin, DE

^9^Illumina Cambridge Ltd., Chesterford Research Park, Little Chesterford, UK

^10^Department of Histopathology, Royal National Orthopaedic Hospital NHS Trust, Stanmore, Middlesex, UK

*corresponding author: SB <s.beck@ucl.ac.uk>

**Supplementary Table 1: Final MeDIP-seq read counts for osteosarcoma samples**

The fastq files were aligned to both Human (hg19) and Mouse (mm10) genomes. Only reads that aligned to either Human-only or both Human and Mouse were retained for downstream analysis.

**Supplementary Table 1**
